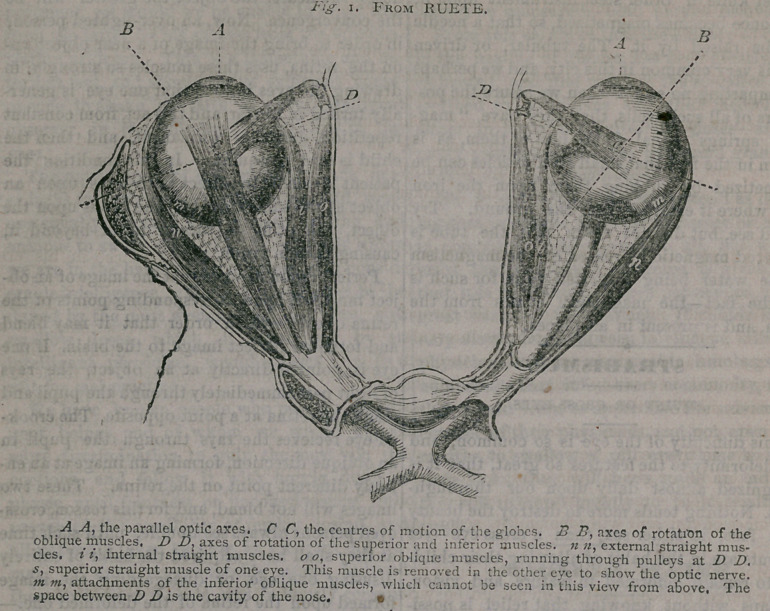# Strabismus

**Published:** 1872-10

**Authors:** 


### STRABISMUS.

SQUINT OR CROSS EYES.

This difficulty of the eye is so common, and the deformity to the features so great, that it is recognized almost daily upon our thoroughfares. Nothing tends more to destroy the beauty of a face, in either sex, than a cross-eye—yet parents are in the habit of allowing their children to remain deformed in feature and vision, perhaps without knowing that relief is possible.

Childrens' eyes are made to squint from several causes, prominent among which are diseases of the brain, common to infantile life; the habit of looking at objects in close proximity to the face, or at the end of the nose; paralysis of one or more muscles of the eye; spots upon the cornea, requiring the eye to turn in or out in order that light may enter the pupil one side of the obstruction; attempts at imitating the squint of persons so afflicted, etc. The usual cause for *convergent* squint, (that form in which the crooked eye turns towards the nose), is an abnormal shortening of the ball of the eye, from in front backwards, a condition which produces "over-sightedness," or what ophthalmic surgeons term *hypermetropia.* In this condition of the eye the rays cannot be focused upon the retina, they fall too far behind this point, and can only be made to form an image on the retina by means of an excessive effort of the muscles of the eye, used in *accommodation;* or, in other words, without great strain upon those muscles which adjust the eye to near objects.

It will be observed that when the eyes are accommodated for objects close to the face, that both eyes are turned more or less towards the nose—the nearer the object the greater will be the convergence. Now, an over-sighted person, in order to bring the image of a near object upon the retina, uses these muscles so strongly, in drawing the eyes inward that one eye is generally turned in too far, and this act, from constant repetition, becomes permanent, and then the child is said to squint. In this condition the patient is unable to direct both eyes upon an object at the same time; if one eye is upon the object, the other is turned in or beyond it, causing double vision.

Perfect vision requires that the image of an object must fall upon corresponding points of the retina of each eye, in order that it may blend and form *one* perfect image to the brain. If one eye is pointed directly at an object, the rays of light pass immediately through the pupil and reach the retina at a point opposite. The crooked eye recieves the rays through the pupil in an oblique direction, forming an image at an entirely different point on the retina. These two images will not blend, and for this reason, cross-eyed persons always see double, until such time as they shall have acquired the habit of entirely disusing one eye, or disregarding the image formed upon the retina of the deformed one.—This is very soon learned by the squinting person, in the same manner that the watch-maker or microscopist learns to look through a magnifying glass or microscope without closing one eye; they soon learn to disregard the image formed upon the retina of the eye not used, and so does the person suffering from cross-eye.—But if this habit should be presisted in every hour in the day, and every day in the year, as must be the case with the squinting person, the eye that is allowed to remain in disuse soon becomes greatly impaired, and finally entirely blind. For this reason, a child should be operated upon while young—say from three to four years of age—before the habit of suppressing the image has been practiced long enough to permanently injure the vision.

If the strabismus is caused by *hypermetropia,* or over-sightedness, it can always be remedied by the use of properly adjusted glasses, *if applied in time.* But if the case is neglected, with a hope that the child may "out grow it," the squint becomes permanent, the inner straight-muscle of the eye becomes shortened, the outer one weakened, so that the eye can no longer be pulled outward, when the following operation must be performed to afford relief and render both eyes straight:

In order that the operation may be properly understood, and that it may be seen what a simple and harmless one it is—if skillfully performed—we give the above excellent engraving of the two eyes, natural size and shape, showing their external anatomy, and in addition to the description we add the following explanation:

A A are the corneæ, and represent the direction in which the eyes are supposed to be looking. The dotted lines D D, show the distance that the eyes are capable of moving from right to left; n n are the two outer straight muscles, which move the eye outward; i i are the two inner straight muscles, used in drawing the eye toward the nose; O O are the two upper oblique muscles which rotate the eye upon the axis B, and also, in unison with the lower oblique muscle, elongates the globe. In the left eye, s is the upper straight muscle, which rolls the eye upward; in the right eye it is removed to exhibit the optic nerve which lies under it. The lower straight muscle, of course, cannot be seen, as we are looking upon the eyes from above, downward. The point where the two optic nerves are seen to cross and intermingle is within the skull. The operation for cross-eye now will be plain, when we explain that the tendon of the internal straight muscle i, is cut at the point where the dotted line D crosses it in the above figure. The muscle, when severed, draws back and attaches itself again to the globe, while the eye so operated upon, turns out to correspond with its fellow. The well eye is then bandaged for a few days, throwing all the labor on the one operated upon, so insuring its straight position while the cut tendon is attaching itself. This is the simple means adopted for the cure of *convergent* squint or inturning of the eye-ball. There exists still another form, called *divergent* strabismus, in which the ball turns outward, and is quite as much of a deformity, both to vision and features, as the convergent form. This is also remedied by cutting the internal straight muscle, as before, and bringing it forward and attaching it to the globe further in front, and sometimes also cutting the external muscle n, where the dotted line D crosses it in the right eye of the figure.

The opinion is widespread that the operation for straightening a cross-eye is a terrible slaughter, in which the eye is removed, nerves and cords cut, and sight ruined. We hope the description here given and the plate illustrating the operation, will be sufficiently clear to dispel all such nonsense, and induce parents to have their little ones seen by an ophthalmic surgeon, in cases of this character, before vision is permanently injured. In these days of chloroform and ether, no fear need be experienced from pain in such a procedure, for an eye can be easily and painlessly straightened, deformity removed and vision restored, in the manner described.

## Figures and Tables

**Fig. I. f1:**